# Supplementary material for: Patterns of Intron Gain and Loss in Fungi
Source: PLoS Biol. 2004 Nov 30;2(12):e422. doi: 10.1371/journal.pbio.0020422 (PMC532390; doi:10.1371/journal.pbio.0020422)
Supplement: Table S1 — Also available at http://genes.mit.edu/NielsenEtAl/. (4.3 MB ZIP). [file pbio.0020422.st001.zip › NielsenEtAl/html/1211.html]

AN6493.1.NCU00117.1.MG00471.1.FG02609.1


```
 CLUSTAL W (1.82) Multiple Sequence Alignments - Introns Inserted


Sequence 1: NCU00117.1	758 aa
Sequence 2: MG00471.1	755 aa
Sequence 3: FG02609.1	771 aa
Sequence 4: AN6493.1	741 aa
Alignment Length: 801 aa
Number Identitical Residues: 343 aa
Alignment Score (without introns) 17876


MG00471.1 	--~---------------------MPRKPQSYD-N~YGAAVPE0IILASSDSDFLDQLIP
NCU00117.1	--~---------------------MPRKVQTWD-D~YDSAVDQ0IILTPSDSDFIDQLIP
FG02609.1 	MD0MLYYLGAGRLNVNVEHQFECCAPFFAQEVASN0ITSPSIE0VILASSDAEFLDQLIP
AN6493.1  	--~---------------------MPSMVPSRS-E~SLYVLNQ0IIISPSDTDYLDQLIP
          	                         *        :       : :*::.**::::*****

MG00471.1 	VLKDASQGGRMGALVQNFSQYAADRESEIEMVGLTKHEEFLGSVNQLQKVREGTVKLTAE
NCU00117.1	VLKDATASGRTATLVQSLSQYAEEREGDIERIGLTQHEEFLGSVSQLQTIREETVALTAE
FG02609.1 	VLKDATHSNRTPLLTQCLSRYYEDREAEIERIGLTRHEEFLDSINHLQTVRAETVTLTQE
AN6493.1  	SIKEYSVGNKTSELLRSLSKFASDKEAEIENICNTNHQEFVSSVNQLLHIREGTVSLTAE
          	 :*: : ..:   * : :*::  ::*.:** :  *.*:**:.*:.:*  :*  ** ** *

MG00471.1 	ILELNQSIQASTEKLAEQKQSLVNTRAVRQNIADASEALKESLKILHAVNNALDLIRKKN
NCU00117.1	ILDLNQSIQASTEKLAEQKQALVDTRRVRQNITDVSDALRESLKIMHAVNNAHDLIRKKK
FG02609.1 	ILSLNESIATSTKKLATQKEALVNTSAVRQNIADATSALNDSLTILRAVNNAHDLVRRKK
AN6493.1  	ILDLNQSIQASTEKLAEQKKALVESRQHRLNIDETSRAIQDCLEVLRLANQVHDLLAKKN
          	**.**:** :**:*** **::**::   * ** :.: *:.:.* ::: .*:. **: :*:

MG00471.1 	YYAALKSLDDLQNEFLIPTIQNKYATQHKLADLIQKSIPAWQKSICDAVMTDLNTWLFRI
NCU00117.1	YYGALKSLEDLQNEYLVPIIQNKYATQYRLADLIQKSIPASRKTISEAVMTDLNTWLYRI
FG02609.1 	YYAALKSLDDLQNEHLVPIIQNRYSTQHRLADVIQKSIPASQKAISEAVMTDLNTWLFRI
AN6493.1  	HYAALRALEELQNVHLKG------VTQFQIADMIQRSVPATQRAIAEAVMSDLNTWLYRI
          	:*.**::*::*** .*         **.::**:**:*:** :::*.:***:******:**

MG00471.1 	RETSQYLGEVAMWHTELRRERQRERVEANDFLKRFRLNSAIELVYDESEEFDVLDNEELQ
NCU00117.1	RETSQFLGEVAFFGTRQRLDRQRERAEQNPYLGHFKLNSAIELVFDESDEFDVLDNEEVQ
FG02609.1 	RETSQFLGEVAFYHTEMRRTRQKKRVEDDPFLANFKLNSAIELVSDENEDFDVLNNEELQ
AN6493.1  	REMSQYLGEIALYHTDLRKTRQKERAARLPYLEHFKLNSAIELVCDEHEEFDLLQNEELQ
          	** **:***:*:: *  *  **::*.    :* .*:******** ** ::**:*:***:*

MG00471.1 	VDFTPLFEAVHIHDALTQVERFKSDYAATRRQQKDLLMPTRVNLEG-EEEPTLSALLEGI
NCU00117.1	VDFTPLHEALHIHEALGQIDKFRAEYAATRRQQKELLMPSSVNLSSDEEENSLRDLLEQI
FG02609.1 	VDFYPLLEALHIHEALGQLDKFRSEYGATRRQQKDLLLPSSIGLLA-EDEQSLSSLLEGI
AN6493.1  	VDFTPLFECLHIHQSLGQMEKFRVEYANTRRRQKELLIPASVTLVD-DDGASLHNLLEEM
          	*** ** *.:***::* *:::*: :*. ***:**:**:*: : *   ::  :*  *** :

MG00471.1 	AGFAIIEKATMQRVPQLRSPVD~VDELWDSLCRSAIALVTKALKDVTNAEILLNMKVRIA
NCU00117.1	TGFAVIEKATIQRAPQLRSTVE~VDELWDSMCQTAIRLISRSLTDVDNAELLLKIKGDIA
FG02609.1 	AGFAIIEKATMQRVPHLRSASD0VEELWDSMCTAVINLTSKGLGDIEDAEALIKIKTIIA
AN6493.1  	AGFAIVERATMKKVPDLRYPVD0VDELWESMCHTAVGLISTALHEVDNAESLLKTKNLIA
          	:***::*:**:::.*.** . : *:***:*:* :.: * : .* :: :** *:: *  **

MG00471.1 	LFIQTME~GWGYSVSVLDNFLLTLFDKYAELLKRRFSEDFQE0IVSTDDYMPMAINNVEE
NCU00117.1	LFIQTME0SWNYSVSTLNNFQLTLFYKYAELLKRRFSDDFQE~IVSTDDYMPMQINNAEE
FG02609.1 	LFIQTME0GWGYSVTTLDNFLLKLFDKYAELLKKRFSEDFHE0IVSTDDYMPMAINNPEE
AN6493.1  	LFMQTMN0TWDFAMGAFEDLLLTLFKKYAELLKKRFSDDFQE0IVSTDDYMPMPIQTPEE
          	**:***:  *.::: .:::: *.** *******:***:**:* ********** *:. **

MG00471.1 	YEKVVNVSWFTPEKPAEELS2FPCVLPFSQMYPLCCIDIRNFLNQFYFFSDDHFQHPNII
NCU00117.1	YEKVLKVSWYTEEKAPEELT2-------------------NFLNQFYFFSNDHFQHPNIV
FG02609.1 	YEKVVNVSWFSQEKPTDQIT2YPCVLPFSQMYPLCCIDIRNFLNQFYFFSDDHFQHPTVI
AN6493.1  	FDKVLNVSWYTPSEP-QEQA2FPCVLPFSQMYPLCCIDIRNFLNQFYFFANDDFTNPDII
          	::**::***:: .:. :: :  ..  . :.  . .. .  *********::*.* :* ::

MG00471.1 	DETLRK0SLDSLLTEK0--~-----------------ILINLEHFEIACQELEQLLIRAR
NCU00117.1	DETLRK0SLDELLTEK~VC~RTLVERLNSQYLGQIVQILINLEHFEEACHQLEQELIRAR
FG02609.1 	DETLRK0SLDELLTEK~VC1-------------QIVQILINLEHFEIACRELEQLLIRAR
AN6493.1  	DATLKD0ALDELLSSK~VC~DTLVERLNSQYLGQIVQILINLEHFEHACHELELLLAAAR
          	* **:. :**.**:.*  .  :  .  .:.  ..  .********* **::**  *  **

MG00471.1 	SSTSAGGPVTLSATEQFRSNKKTAEKRIFELVNSKIDDLVDTAEYEW2TAANPQLEPSNF
NCU00117.1	SSTSAGGPVSLKSTEEFRSNKKTAEKRIFELVNSKIDDLVDTSDYNW2MTPSKPTEPSNY
FG02609.1 	SSTSAGGPVTLKATDSFRDNKKTAEKRIFELVNSKIDDLIDTAEYEW2TASTVEKEPSNY
AN6493.1  	SQNFSSEPVALKATGKFRDNKKAAEKRIFEVVNSKIDDLIETAEYDW~TAAAAPTEPSNY
          	*.. :. **:*.:* .**.***:*******:********::*::*:*  :.    ****:

MG00471.1 	IQTLTRYLANIMSSTLLGLPREIKELIYFDALSHAADKIL0TLPMAPEVKRINPNGVQAM
NCU00117.1	MQTLTRYLENIMGSTLLGLPREIKELIYFDALSHAANKIL0ALPLSPDVKKINANAVAAM
FG02609.1 	MQTLTRYLSNIMNSTLLGLPREIKELIYFDALSHTAEKIL0ALPLSNDVKHINPNGVAAL
AN6493.1  	MQTLTRFLSNIMNSTLLGLPKEIKELIYFDALSHASTMIL0ALPLSAEVKRINPNGVMAL
          	:*****:* ***.*******:*************::  ** :**:: :**:**.*.* *:

MG00471.1 	ETDVRYLTEFVESLDNSFMLTQTLDELQQTVELMKSGNSDEFFEAAVRNKKYPRVNAMNG
NCU00117.1	AMDVQHLSAFVANLENAPMLEQNLDELQQTIALMQSDNHDEFFDISTRNKKYGRVDAMNG
FG02609.1 	AQDVDYLVQFVSSLENGQMLRENLDELSQTISLLQTDNQDEFFDISTRNKKYGRVNALNG
AN6493.1  	AKDVEYLYQFVDSLNN-PILRENLDELQQTVQLMQAENADEFYDISMRNKKYGRVDAING
          	  ** :*  ** .*:*  :* :.****.**: *::: * ***:: : ***** **:*:**

MG00471.1 	PILLEK2LSTTVEN--TPKGTPALAGFSSRFGFS
NCU00117.1	PILLEK2LTLGIESTTTSRAAAPLANFGSRFGLR
FG02609.1 	PMLLEK~YDQPICCQVWN----------------
AN6493.1  	PTLLEK~---------------------------
          	* ****
```
